# Development of a bio-electrospray system for cell and non-viral gene delivery

**DOI:** 10.1039/c7ra12477e

**Published:** 2018-02-09

**Authors:** Myung Chul Lee, Hoon Seonwoo, Pankaj Garg, Kyoung Je Jang, Shambhavi Pandey, Hong Bae Kim, Sang Bae Park, Jong Beom Ku, Jang Ho Kim, Ki Taek Lim, Jong Hoon Chung

**Affiliations:** Department of Biosystems & Biomaterials Science and Engineering, Seoul National University Seoul 151-742 Republic of Korea jchung@snu.ac.kr; Department of Industrial Machinery Engineering, Sunchon National University 315 Maegok-dong Suncheon Republic of Korea; Research Institute for Agriculture and Life Sciences, Seoul National University Seoul 151-921 Republic of Korea; Department of Biosystems Engineering, College of Agricultural and Life Sciences, Kangwon National University Chuncheon 200-701 Republic of Korea; Department of Rural and Biosystems Engineering, Chonnam National University Gwangju 500-757 Republic of Korea

## Abstract

Bio-electrospray technology is a very attractive tool for preparing scaffolds and depositing desired solutions on various targets by electric force. In this study, we focused on the application of a bio-electrospray (BES) technique to spray cells on the target and to simultaneously deliver genetic constructs into the cells, called non-viral gene delivery-based bio-electrospray (NVG-BES). Using this method, we tried to harvest the electric charge produced during electrospray for the cellular internalization of cationic polymer/DNA nanoparticles as well as the delivery of living cells on the desired substrate. Furthermore, we optimized the voltage, culture medium and polymeric cationic charges for high transfection efficiency and cell viability during NVG-BES. As a result, the solutions used during the NVG-BES process played an important role in improving transfection efficiency. We determined that a voltage of 10 kV with PBS as the spraying solution showed high transfection efficiency, probably due to the facilitation of cationic polymer/DNA nanocomplexes in cellular internalization and their subsequent expression. In conclusion, NVG-BES, as a novel method, is expected to deliver genes to cells and simultaneously deliver transfected cells to any substrate or scaffold.

## Abstract 2

The NVG-BES system facilitated to introduce DNA to cells and delivered cells to a target simultaneously. In this method, a cationic polymer was used as non-viral carrier with electric force by bio-electrospray (BES) system to electrospray living cells onto a target.

## Graphical abstract

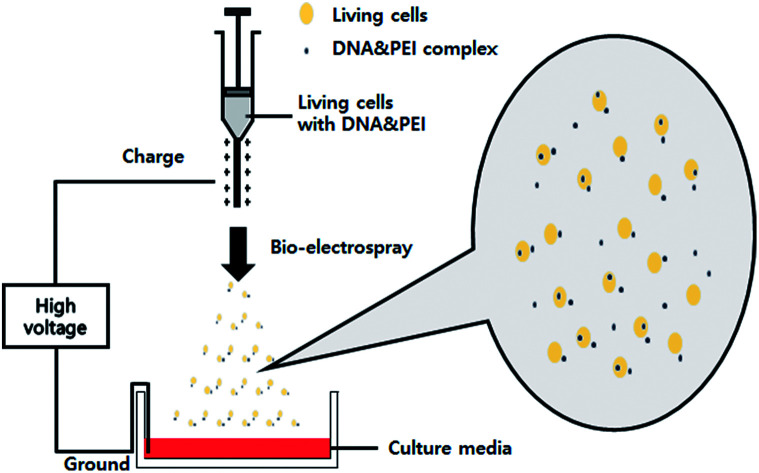

## Introduction

Gene therapy is an emerging means of correcting genetic disorders at the molecular roots, redefining and revolutionizing the practice of medicine in the near future. The clinical use of these therapeutic agents is severely hampered by the lack of an appropriate carrier system to help DNA reach the target cells. Thus far, various viral carrier systems, such as retrovirus and adenovirus carriers, and non-viral carriers, such as cationic lipids, liposomes and nanoparticles, have been exploited for this purpose. Despite the natural ability of viruses to infect host cells, the risk of immunogenicity and the random integration of vector DNA into host chromosomes are associated problems. Therefore, although the transfection efficiencies of non-viral gene delivery systems are low, they have been widely used as safe gene delivery agents.^1,2^ Many researchers have investigated non-viral vectors, including synthetic polymers such as polyethylenimine (PEI), poly(l-lysine) (PLL), polyamidoamine (PAMAM) dendrimers and poly(2-dimethyl amino ethyl)methacrylate (PDMAEMA).^3,4^ Several groups also tried to apply mechanical factors as substitutes for viral and non-viral cationic polymer-based gene delivery methods.^1^ Among physical methods such as direct injection, electroporation, and ultrasound-mediated transfection, electroporation is a typical transfection technique that introduces DNA or drugs into cells by increasing the permeability of the cell membranes using electrical fields.^5^ Based on these methods, a new technique for transfection called electrospray (ES) was developed by Okubo *et al.*^6^ This team showed that the impact by an electrosprayed droplet can be applied to enhance transfection. In the electrospray method, the efficiency of transfection to attached cells was influenced by the charge densities of plasmid-containing sprayed droplets. The size of the droplets could also be an important factor in determining transfection efficiency (Ikemoto *et al.*).^7^ However, the major limitation of electrospray is its inapplicability to non-adhesive or floating cells. Thus, a more convenient method for delivering genes (regardless of cell type) should be developed.

**Fig. 1:**
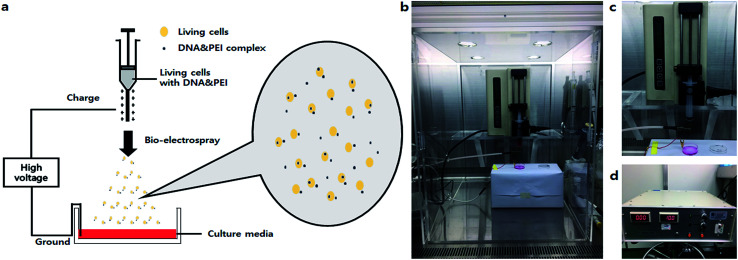
Non-viral gene delivery-based bio-electrospray (NVG-BES) system. (a) Schematic diagram of the NVG-BES system. The NVG-BES system facilitated introduction of DNA to cells and simultaneously delivered cells to a target. In this method, a cationic polymer was used as non-viral carrier with electric force in a bio-electrospray (BES) system to electrospray living cells onto a target. (b) NVG-BES system on a clean bench. (c) The syringe pump. (d) The high voltage generator.

Bio-electrospray (BES) is a technique to deliver cells by the electrospray method on specific targets.^8^ Although some researchers have proposed risks for BES, Sahoo *et al.* clearly demonstrated that the viability of bone-marrow derived mesenchymal progenitor/stem cells (BMSC) was not affected by BES.^8^ Hall *et al.* evaluated the genetic safety of BES on human cells. Bio-electrosprayed (BESed) cells showed minor damage relative to the negative control (BES without the application of voltage).^9^ In addition, Eliot *et al.* reported a transfection method using BES.^10^ However, they used a viral vector as the gene carrier. Viral vector-based gene delivery has potential risks for cells, such as infection or immunogenicity. Thus, we hypothesized that BES with a non-viral vector not only improves transfection efficiency by electric force, but also delivers the cells safely. Herein, we developed a novel method to increase the transfection efficiency and to deliver cells, called non-viral gene delivery-based bio-electrospray (NVG-BES) (Fig. 1). Branched 25 kD polyethyleneimine (PEI), a well-known cationic polymer used in gene delivery, was used in this study to aid gene transfection due to its well-known properties, such as high availability and high DNA complexation ability.^11,12^ NIH-3T3, a murine fibroblast cell line, was also used in this study. We tried to find the optimal NVG-BES conditions for enhancing the transfection efficiency by checking cell viability after NVG-BES.

## Results and discussion

**Fig. 2:**
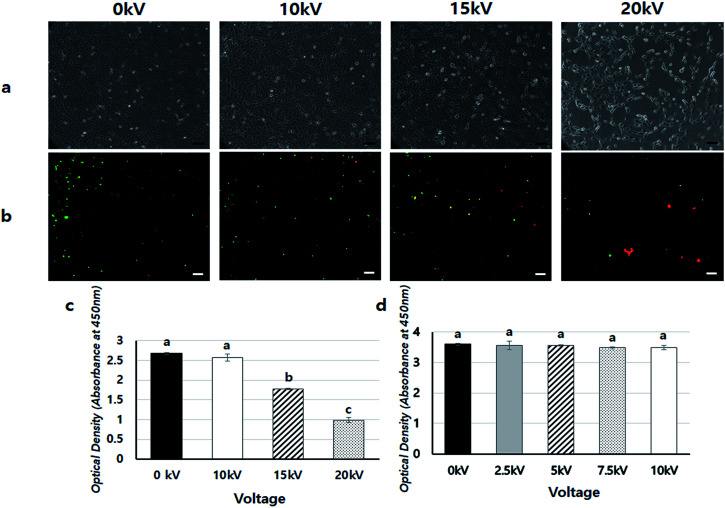
Cell viability assay: (a) cell morphology 1 day after BES at 0 kV, 10 kV, 15 kV, and 20 kV. (b) The results of the live/dead assay 1 day after bio-electrospray at 0 kV, 10 kV, 15 kV, and 20 kV (green: live cells, red: dead cells). The case of 0 kV is the negative control (no BES). The pictures were taken at 10× magnification. (scale bars = 100 μm), (c) WST assay of cultured cells 1 day after BES from 0 kV to 20 kV. The case of 0 kV is the negative control (no BES) (*n* = 3, *p* < 0.05. Columns with different letters are significantly different according to the Duncan test). (d) WST assay of cultured cells 1 day after BES from 0 kV to 10 kV. The case of 0 kV is the negative control (no BES). (*n* = 3, *p* < 0.05. Columns with different letters are significantly different, according to the Duncan test). The results of cell viability assay after BES showed that cells were fine under conditions at 10 kV of BES. However, cell viability was rapidly decreased above 10 kV of BES.

To confirm the safety of BES, we first investigated the cell viability of BES at various voltages (0, 10, 15, and 20 kV) without PEI. Fig. 2a shows the cell morphology 1 day after BES. All groups successfully deposited cells on tissue culture-polystyrene (TCPS). The cell densities at 0 and 10 kV appeared high, but the cell density at 15 kV was lower than that at 10 kV. The cells sprayed at 20 kV had severely low population, which indicates that the cell morphologies appeared fine at 10 kV, while above 10 kV cells appeared damaged and their adhesion decreased. The live/dead assay also showed a similar result (Fig. 2b). The number of red spots (dead cells) appearing on the BESed cells increased with on increasing the applied voltage. Next, we quantitatively measured cell viability using a WST-1 assay (Fig. 2c and d). The viability of sprayed cells decreased remarkably at high voltages (15 and 20 kV groups). However, cell viabilities in the 10 kV group were not significantly different from those at 0 kV. For more detailed analysis, we repeatedly carried out WST assays of cells BESed from 0 to 10 kV (0, 2.5, 5, 7.5, and 10 kV). There was no significant difference between the groups, indicating that values below 10 kV are applicable to BES.

**Fig. 3:**
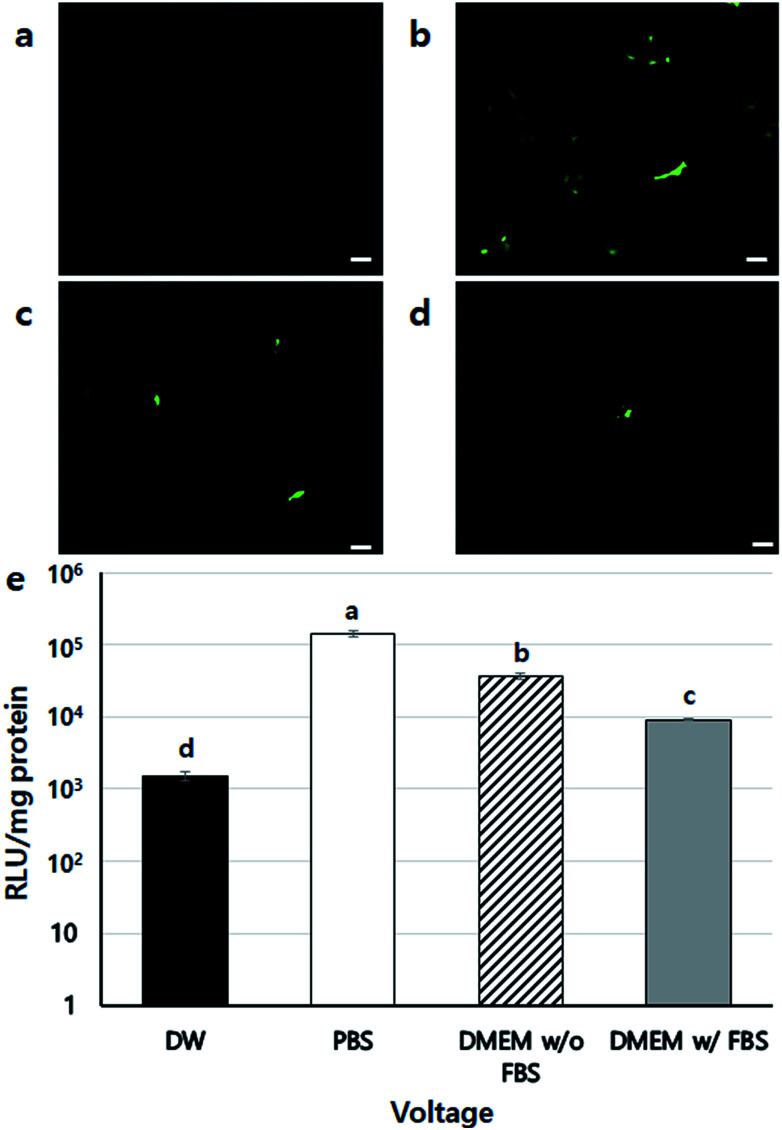
DNA expression results using NVG-BES with several solutions (a)–(d) GFP expression after NVG-BES transfection with several solutions: (a) DW, (b) PBS, (c) DMEM (w/o FBS), (d) DMEM (w/FBS). GFP expression pictures were taken at 10× magnification (scale bars = 100 μm). (e) Luciferase activity after BES with several solutions (*n* = 3 *p* < 0.05. Columns with different letters are significantly different, according to the Duncan-test). Other groups were significantly different to the DW group (*p* < 0.001). The use of PBS as BES solution showed higher expression of GFP than the other solutions.

Next, the gene transfection ability of NVG-BES was investigated. To verify whether NVG-BES increased the transfection efficiency with or without using a polymer carrier, optimal NVG-BES conditions were investigated. First, we tested several solutions such as phosphate buffered saline (PBS), deionised water (DW), DMEM without foetal bovine serum (DMEM w/o FBS) and DMEM with FBS (DMEM w/FBS) to determine the appropriate solution for NVG-BES. The transfection efficiency was verified *via* green fluorescence protein (GFP) and luciferase assays. The NVG-BES was performed at 10 kV because there was no significant difference in cell viability below 10 kV. The PBS group showed better green fluorescence during the GFP assay than the other groups, while the DW group showed very low expression of GFP (Fig. 3a–d). The luciferase assay also showed that the PBS, DMEM w/FBS, and DMEM w/o FBS groups exhibited significantly higher transfection levels (*p* < 0.001) than the DW group. Furthermore, the PBS group exhibited the highest luciferase expression level (Fig. 3e). The GFP and luciferase assays both showed that the best solution for NVG-BES was PBS. The NVG-BES results were affected by the type of solution, that is, the solution used is an important factor in NVG-BES. The DMEM w/FBS group was anticipated to produce lower transfection efficiency than the DMEM w/o FBS group because serum usually interferes with binding between DNA and polymer.^13–15^ As expected, the DMEM w/o FBS group exhibited significantly higher luciferase gene expression than the DMEM w/FBS group. In addition, the DW group showed the lowest luciferase gene expression. Because cells cannot bear the osmotic pressure when resuspended in DW after harvesting, the cell viability decreased rapidly, resulting in hard gene transfection. Unlike DW, PBS contributes to cell viability as a buffer, helping to maintain cell conditions. Chesnoy *et al.* suggested that PBS helps stabilize DNA against degradation.^16^ PBS was also used by Roos *et al.* as a DNA vehicle solution for electroporation and by Kang *et al.* as a buffer for nucleofection (an application of electrophoresis).^17,18^ Thus, PBS was the best solution and displayed the highest transfection efficiency for NVG-BES.

**Fig. 4:**
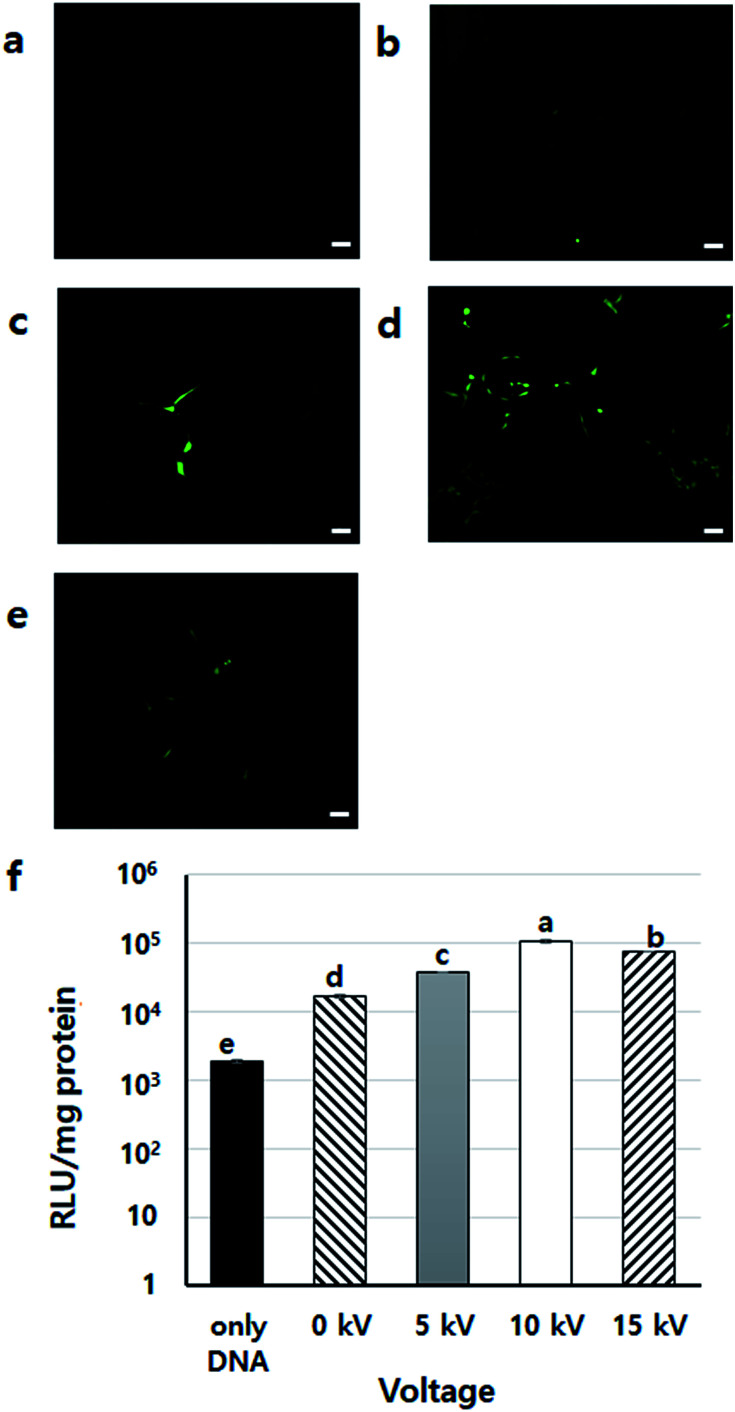
DNA expression results using NVG-BES at various voltages (a)–(f) GFP expression results after bio-electrospray at different voltages: (a) only DNA, (b) 0 kV, (c) 5 kV, (d) 10 kV and (e) 15 kV. The 0 kV group received only PEI + DNA (no BES), and the case labelled only DNA received only DNA. GFP expression pictures were taken at 10× magnification (scale bars = 100 μm). At 10 kV of BES, GFP was expressed more frequently than other groups. (f) Luciferase assay results after BES at different voltages (*n* = 3, *p* < 0.05. Columns with different letters are significantly different according to the Duncan test). Other groups were significantly different (*p* < 0.001) than the only DNA group (N.C). The groups using PEI and those applying BES had increased expression of luciferase. In particular, BES at 10 kV had the highest expression level.

To find the appropriate voltage, cells with DNA/PEI complex were BESed at various voltages (0, 5, 10, and 15 kV). From the GFP expression results, the 10 kV group had more green-fluoresced cells than the other groups (Fig. 4a–e). In the luciferase assay, compared to the only DNA group, all groups showed significantly higher transfection levels (*p* < 0.001) (Fig. 4f). In particular, the gene expression of the 10 kV group was significantly higher than those of the other groups, and the luciferase activity of the 10 kV group was approximately 6 times higher than that of the 0 kV group. Although the gene transfection level of the 15 kV group was also high, cell viability of the 15 kV group decreased severely. The gene transfection level of the 15 kV group was expected to decrease due to cell death induced by excessively high voltage. However, an appropriate voltage may increase the transfection efficiency of NVG-BES. As the cell viability of the 10 kV group was not severely lower than that of the 15 kV group, the appropriate voltage for NVG-BES for transfection was fixed at 10 kV.

**Fig. 5:**
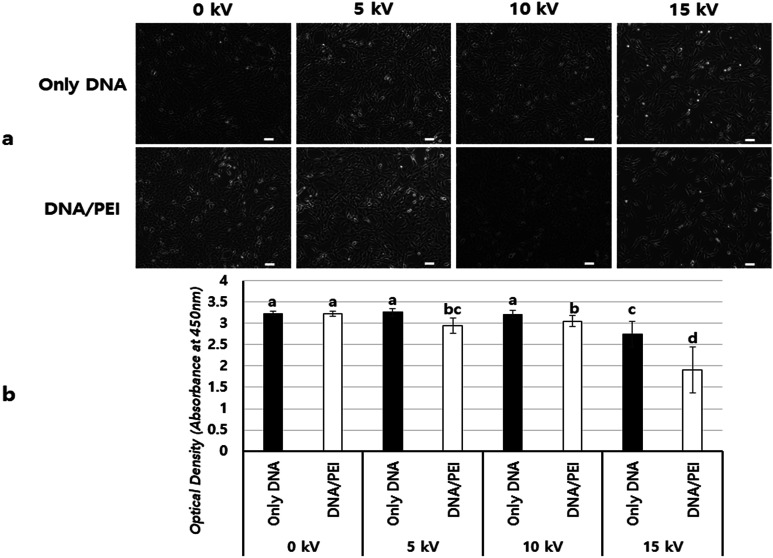
Comparison of viability between BES with only DNA and BES with PEI & DNA (a) image of BESed cells after one day at 0 kV, 5 kV, 10 kV and 15 kV. The pictures were taken at 10× magnification (scale bars = 100 μm). (b) WST-1 results (*n* = 7, *p* < 0.05. Columns with different letters are significantly different, according to the Duncan test). The case of 0 kV is no BES. However, cell morphology showed no difference at 10 kV of BES, and cell viability of BES with PEI decreased significantly relative to groups of BES without PEI.

**Fig. 6:**
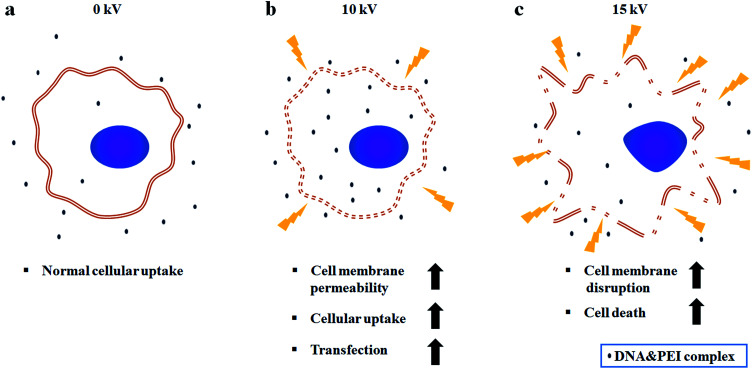
Proposed NVG-BES mechanism. (a) At 0 kV, normal cellular uptake occurs. (b) At 10 kV, cell membrane permeability and cellular uptake are increased by induction of electric field. As a consequence, transfection efficiency increases. (c) Above 10 kV, cell membrane disruption and cell death happen due to excessive electric field.

Fig. 5 shows a comparison of cell viabilities after NVG-BES with only DNA and with DNA/PEI at various voltages (0, 5, 10, and 15 kV). Although there were no noticeable morphological differences between the cases with PEI and without PEI (Fig. 4a), the results of the WST-1 assay showed that cell viability in the DNA/PEI group was significantly lower in comparison with the only DNA group when voltages were induced (Fig. 4b). Interestingly, the cell viability of the voltage-induced DNA/PEI group decreased even though there was no significant difference in the cell viability of the only DNA group. It is expected that the change in electric force induced by NVG-BES stimulates the delivery of DNA/PEI complexes into cells, resulting in cytotoxicity. For the relevant application of NVG-BES, finding the appropriate voltage introduced DNA and also minimized the cytotoxicity. Fig. 6 shows proposed NVG-BES mechanisms. Electric shocks were reported to enhance the permeability of the cell membrane, resulting in electroporation.^5,19–21^ When the electrospray generates electric fields,^22,23^ similar to the electroporation method, the electric fields generated between the needle and culture dish are anticipated to increase cell permeability, leading to increased cellular uptake and transfection efficiency. To reveal the underlying mechanism thoroughly, more studies are needed.

**Fig. 7:**
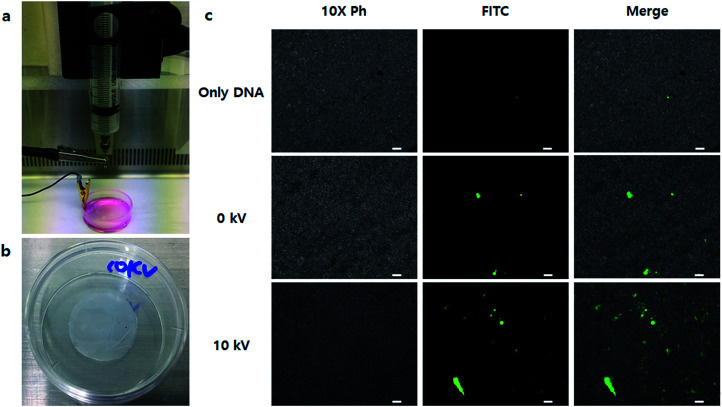
Delivery transfected cells on the scaffold using the BES transfection method. (a) Bio-electrospray on a PCL nanofiber scaffold. (b) The PCL nanofiber scaffold. (c) The results of delivering transfected cells on a PCL nanofiber scaffold using BES. GFP expression pictures were taken at 10× magnification (scale bars = 100 μm). It showed that BES transfected living cells and delivered them to a nanofiber scaffold at the same time.

Finally, we applied the NVG-BES to nanofibrous scaffolds to induce simultaneous cell delivery and gene transfection. After nanofibrous scaffolds prepared by the electrospinning method were deposited on a 35 mm dish, NIH-3T3 cells were BESed onto the nanofibrous scaffolds with a GFP gene or GFP gene/branched 25 kD PEI complex at 0 and 10 kV (Fig. 7a and b). As a result, cells were not only delivered onto nanofibrous scaffolds, but also transfected in the 10 kV group (Fig. 7c). Our study suggests a new application for BES. NVG-BES not only can deliver cells to any substrate, such as patches or scaffolds, but also transfers genes to cells in the non-adhesive condition at the same time. Furthermore, the method can be used to prepare and deliver induced pluripotent stem cells (iPSCs) from fibroblasts with Yamanaka factors. If it can turn fibroblasts extracted from patients directly into stem cells, a novel treatment technique in stem cell therapy could be presented. However, NVG-BES with PEI is cytotoxic to cells. Thus, we will improve the BES method by applying a less toxic cationic-polymer than 25 kD PEI. Although it is hard to control BES conditions, this technique can be used as a new transfection method and applied in tissue engineering.

## Conclusions

In summary, we developed a novel NVG-BES system that can simultaneously deliver cells and induce gene transfection. First, we confirmed the voltages suitable for cell viability after BES. Voltages below 10 kV were appropriate for BES due to the resultant good cell viabilities. Second, the cell viability of BES with PEI decreased significantly in comparison with the groups with BES without PEI. Third, we discovered that PBS is the best solution for BES and BES at 10 kV has the highest gene transfection efficiency. Finally, it was possible to directly seed transfected living cells on scaffolds using the BES technique.

## Materials and methods

### Cell preparation

NIH-3T3 (ATCC, USA) cells were cultured in a T75 flask (NUNC) for 2 days with Dulbecco's Modified Eagle's Medium (DMEM, Welgene Inc., Republic of Korea) containing antibiotics (Welgene Inc., Republic of Korea) and 10% foetal bovine serum (FBS, HyClone, USA). NIH-3T3 cells were seeded at a density of 2 × 10^6^ cells per mL. After 2 days, the cells were harvested.

### Bio-electrospray

The non-viral gene delivery-based bio-electrospray (NVG-BES) system was set on a clean bench. The NVG-BES system consisted of two main components: syringe pump (KDS100, 789100, kdScientific, USA) and high voltage generator (DC(+)TKM45K5M, TEKKAM, Republic of Korea). The solution of harvested cells with culture medium was placed in a 10 mL syringe. Then, the syringe was fixed on a syringe pump. Next, a new 35 mm dish (NUNC, USA) was filled with culture medium and placed on a support. The cell suspension in the syringe was then electrosprayed using a syringe pump and a high voltage supply at various voltages between the syringe tip and a grounded clip on the 35 mm dish. The cell suspension was electrosprayed through a needle equipped to the syringe at a flow rate of 10 mL h^−1^. The electrosprayed cells were dropped on a target 35 mm dish filled with culture medium. The distance between the syringe tip and the grounded clip was 3 cm. Electrospray was performed at room temperature.

### Observation of cell morphology

One day after bio-electrospray, the cell morphology was observed using a microscope (Ti-E, Nikon, Japan). The cell density for this BES experiment was 5 × 10^5^ mL^−1^ and the voltage of the electrospray was varied (0 kV, 10 kV, 15 kV, and 20 kV).

### Live/dead assay

To investigate cell viability after BES with PEI/DNA, a Live/Dead Cell Assay kit (Abcam ab115347, Mitosciences, USA) was used after 1 day of bio-electrospray with a branched 25 kD PEI/DNA complex. When bio-electrospray was run on the dish, the density of cells was 5 × 10^5^ mL^−1^ and the electrospray voltage was varied (0 kV, 10 kV, 15 kV, and 20 kV). The concentration of provided live/dead dye was 1000×. When dye was used for the assay, it had to be diluted 5× in PBS. The cells were stained with 5× live/dead dye and incubated 20 minutes at room temperature. Then, the dyed cells were observed by fluorescence microscopy (Ti-E, Nikon, Japan).

### Cell viability assay after BES

To measure cell viability quantitatively, a WST assay was carried out after bio-electrospray. The cell density was 5 × 10^5^ mL^−1^ and the electrospray voltage was varied (0 kV, 10 kV, 15 kV, 20 kV). One day after electrospray, cell viability assay reagent (EZ-cytox, EZ-3000, DOGEN, Republic of Korea) was added to each cell culture dish and the culture medium in the dish and the reagent were allowed to react for 3 h. Then, each sample was quantified using a Sunrise TM absorbance reader (TECAN, Switzerland) at 450 nm. In addition groups with voltage up to 10 kV (0 kV, 2.5 kV, 5 kV, 7.5 kV, and 10 kV) were run by the same method to verify the results.

### Transfection by NVG-BES

Transfection by bio-electrospray was performed using several mixing solutions. NIH-3T3 cells were cultured in a T75 flask (NUNC) for 2 days with DMEM containing antibiotics and 10% FBS. NIH-3T3 cells were seeded at a density of 2 × 10^6^ cells per mL. After 2 days, various BES mixing solutions were tested for transfection efficiency when the cells were harvested and electrosprayed: phosphate buffer saline (PBS, Welgene Inc., Korea), deionized water (DW), DMEM, and DMEM with serum. The cells with various mixing solutions (500 μL) and branched PEI25kD/DNA polyplexes (200 μL) were mixed and placed in a 10 mL syringe. The branched PEI25kD/DNA polyplexes were used at a 15 N/P ratio. The 25 kDa branched PEI was purchased from Sigma Aldrich (408727-100ML, Sigma, USA). The solution was electrosprayed using a syringe pump and a high voltage supply at 10 kV between a syringe tip and a grounded clip on a 35 mm dish filled with culture medium. The amount of cells electrosprayed was 5 × 10^5^ mL^−1^. As soon as electrospraying was complete, fresh culture medium (1 mL) was added to the dish.

### Cell viability assay after NVG-BES

NIH-3T3 cells were seeded at a density of 2 × 10^6^ cells per mL. After 2 days, the cells were harvested and electrosprayed with only DNA or a DNA-PEI complex in PBS at several voltages. The harvested cells with PBS (500 μL) and branched PEI25kD/DNA polyplexes (200 μL) or only DNA solution (200 μL) were mixed and placed in a 10 mL syringe. The branched PEI25kD/DNA polyplexes were used at a 15 N/P ratio. The cell suspension with only DNA or DNA/PEI complex was electrosprayed using a syringe pump and a high-power supply at various voltages (0 kV, 5 kV, 10 kV, and 15 kV) between the syringe tip and a grounded clip on a 35 mm dish filled with culture medium. The amount of cells electrosprayed was 5 × 10^5^ mL^−1^. As soon as the electrospraying was complete, fresh culture medium (1 mL) was added to the dish. After 1 day, the cell morphology was observed by microscope (Ti-E, Nikon, Japan) and cell viability was measured using a WST-1 assay kit (EZ-cytox, EZ-3000, DOGEN, Republic of Korea).

### Transfection at different voltages of NVG-BES

NIH-3T3 cells were cultured in a T75 flask (NUNC) for 2 days with DMEM containing antibiotics and 10% FBS. NIH-3T3 cells were seeded at a density of 2 × 10^6^ cells per mL. After 2 days, various electrospray voltages were tested for transfection efficiency. Phosphate buffer saline (PBS, Welgene Inc., Republic of Korea) was used as the BES solution. The solutions of harvested cells in PBS (500 μL) and PEI25k/DNA polyplexes (200 μL) were mixed and placed in a 10 mL syringe. The solution was electrosprayed using a syringe pump and a high-power supply at several voltages (0 kV, 5 kV, 10 kV, and 15 kV) between the syringe tip and a grounded clip on a 35 mm dish filled with culture medium. The cell density of the electrosprayed cells was 5 × 10^5^ mL^−1^. As soon as electrospray was complete, fresh culture medium (1 mL) was added to the dish.

### The fabrication of PCL nanofiber scaffold

The PCL nanofiber scaffold was fabricated using poly(ε-caprolactone) (PCL, MW: 80 000, Sigma Aldrich, USA) and the electrospinning technique. To prepare the polymer solution for electrospinning, PCL was dissolved in 2,2,2-trifluoroethanol (TFE) (ReagentPlus® ≥99%, *M*_w_ = 100.04 g mol^−1^, Sigma Aldrich, USA) at 16% weight/volume and acetic acid (F.W. 60.05, Duksan, Republic of Korea) was added at 2% v/v. Fiber was collected at 18 kV (1.8 kV cm^−1^) and syringe pump rate of 0.6 mL h^−1^ for 30 min. The electrospinning scaffold was attached to the PDMS layer. Then, 20 mm diameter samples were obtained by punching, washed with 70% EtOH, and then washed with DPBS three times before BES. Then, the nanofiber scaffold samples were attached on 35 mm dishes.

### The delivery of transfected living cells by NVG-BES

NIH-3T3 cells were cultured in a T75 flask (NUNC) for 2 days with DMEM containing antibiotics and 10% FBS. NIH-3T3 cells were seeded at a density of 2 × 10^6^ cells per mL. After 2 days, BES was applied on a PCL nanofiber scaffold. Phosphate buffer saline (PBS, Welgene Inc., Republic of Korea) was used as the BES solution. The solutions of harvested cells in PBS (500 μL) and PEI25k/DNA polyplexes (200 μL) were mixed and placed in a 10 mL syringe. The solution was electrosprayed using a syringe pump and a high-power supply at 0 kV and 10 kV between the syringe tip and a grounded clip on a 35 mm dish containing PCL scaffold filled with culture medium. The cell density of the electrosprayed cells was 5 × 10^5^ mL^−1^. As soon as electrospray was complete, fresh culture medium (1 mL) was added to the dish.

### The turbo green fluorescent protein (tGFP) assay

The green fluorescent protein (GFP) gene was obtained from Clontech (Palo Alto, CA, USA). PEI25K/GFP (4 μg) polyplexes were used at a 15 N/P ratio. After transfection by bio-electrospray using PEI/GFP, cells were cultured for 48 h in a CO_2_ incubator. The transfected cells were then observed by fluorescence microscopy (Ti-E, Nikon, Japan).

### Luciferase assay

Luciferase reporter, pGL3-vector with SV-40 promoter, and enhancer encoding firefly (*Photinus pyralis*) luciferase were obtained from Promega (Madison, WI, USA). PEI25K/pGL3 (4 μg) polyplexes were used at a 15 N/P ratio. After transfection by bio-electrospray using PEI25K/pGL3, cells were cultured for 48 h in a CO_2_ incubator. The luciferase assay was then performed according to the manufacturer's protocol. A multiple plate reader (Victor3, Perkin Elmer, USA) was used to measure relative light units (RLUs) (normalized by protein concentration) in the cell extract estimated using a BCA protein assay kit (Pierce Biotechnology, Rockford, IL, USA).

### Statistical data analysis

The statistical analysis was performed using R v3.2.1 software (The R Foundation, http://www.r-project.org). The least significant difference (LSD) method, Duncan's test, and one-way ANOVA were used to compare the means of the properties of the samples. The level of significance was *p* < 0.05. The data were reported as the mean ± standard deviation, *n* = 3.

## Conflicts of interest

The authors declare no conflict of interest.

## Supplementary Material

## References

[cit1] Kim T. K., Eberwine J. H. (2010). Anal. Bioanal. Chem..

[cit2] Pear W. S., Nolan G. P., Scott M. L., Baltimore D. (1993). Proc. Natl. Acad. Sci. U. S. A..

[cit3] Fischer D., Bieber T., Li Y., Elsasser H. P., Kissel T. (1999). Pharm. Res..

[cit4] Kunath K. (2003). J. Controlled Release.

[cit5] Mehier-Humbert S., Guy R. H. (2005). Adv. Drug Delivery Rev..

[cit6] Okubo Y., Ikemoto K., Koike K., Tsutsui C., Sakata I., Takei O., Adachi A., Sakai T. (2008). Angew. Chem..

[cit7] Ikemoto K., Sakata I., Sakai T. (2012). Sci. Rep..

[cit8] Sahoo S., Lee W. C., Goh J. C., Toh S. L. (2010). Biotechnol. Bioeng..

[cit9] Hall R. P., Ogilvie C. M., Aarons E., Jayasinghe S. N. (2008). Analyst.

[cit10] Ward E., Chan E., Gustafsson K., Jayasinghe S. N. (2010). Analyst.

[cit11] De Smedt S. C., Demeester J., Hennink W. E. (2000). Pharm. Res..

[cit12] Lee B., Chae H., Tuyen T. N., Kang D., Kim H., Lee M., Ihm S. (2009). Int. J. Mol. Med..

[cit13] Kabanov A. V. (1999). Pharm. Sci. Technol. Today.

[cit14] Nchinda G., Uberla K., Zschornig O. (2002). BMC Biotechnol..

[cit15] Kuo J. H. S. (2003). Biotechnol. Appl. Biochem..

[cit16] Chesnoy S., Huang L. (2002). Mol. Ther..

[cit17] Roos A. K., Eriksson F., Walters D. C., Pisa P., King A. D. (2009). Mol. Ther..

[cit18] Kang J., Ramu S., Lee S., Aguilar B., Ganesan S. K., Yoo J., Kalra V. K., Koh C. J., Hong Y. K. (2009). Anal. Biochem..

[cit19] Chang D. C., Reese T. S. (1990). Biophys. J..

[cit20] Neumann E., Schaeferridder M., Wang Y., Hofschneider P. H. (1982). EMBO J..

[cit21] Gehl J. (2003). Acta Physiol. Scand..

[cit22] Deitzel J. M., Kleinmeyer J. D., Hirvonen J. K., Tan N. C. B. (2001). Polymer.

[cit23] Zheng Y. S., Gong R. H., Zeng Y. C. (2015). RSC Adv..

