# Correction: Development of a bio-electrospray system for cell and non-viral gene delivery

**DOI:** 10.1039/c8ra90023j

**Published:** 2018-03-16

**Authors:** Myung Chul Lee, Hoon Seonwoo, Pankaj Garg, Kyoung Je Jang, Shambhavi Pandey, Hong Bae Kim, Sang Bae Park, Jong Beom Ku, Jang Ho Kim, Ki Taek Lim, Jong Hoon Chung

**Affiliations:** Department of Biosystems & Biomaterials Science and Engineering, Seoul National University Seoul 151-742 Republic of Korea jchung@snu.ac.kr; Department of Industrial Machinery Engineering, Sunchon National University 315 Maegok-dong Suncheon Republic of Korea; Research Institute for Agriculture and Life Sciences, Seoul National University Seoul 151-921 Republic of Korea; Department of Rural and Biosystems Engineering, Chonnam National University Gwangju 500-757 Republic of Korea; Department of Biosystems Engineering, College of Agricultural and Life Sciences, Kangwon National University Chuncheon 200-701 Republic of Korea

## Abstract

Correction for ‘Development of a bio-electrospray system for cell and non-viral gene delivery’ by Myung Chul Lee *et al.*, *RSC Adv.*, 2018, **8**, 6452–6459.

The authors regret that in the original version of the manuscript the affiliations of Jang Ho Kim and Ki Taek Lim were reversed. The correct affiliations are presented in this correction notice.

The Royal Society of Chemistry apologises for these errors and any consequent inconvenience to authors and readers.

## Supplementary Material

